# Primary splenic anaplastic variant of diffuse large B-cell lymphoma: a case report 

**DOI:** 10.1186/s13256-021-02846-x

**Published:** 2021-05-06

**Authors:** Sawsan Ismail, Filip Ali, Hussein Ajamieh, Samir Kanaan, Rana Issa, Ali Daoud, Zuheir Alshehabi

**Affiliations:** 1 Department of Pathology, Cancer Research Center, Faculty of Medicine, Tishreen University, Lattakia, Syria; 2 Faculty of Medicine, Tishreen University, Lattakia, Syria; 3 Faculty of Medicine, Tishreen University, Lattakia, Syria; 4 Department of General Surgery, Tishreen University Hospital, Lattakia, Syria; 5 Department of Pathology, Tishreen University Hospital, Lattakia, Syria; 6 Department of Pathology, Tishreen University Hospital, Lattakia, Syria; 7 Department of Pathology, Cancer Research Center, Tishreen University Hospital, Lattakia, Syria

**Keywords:** Primary splenic lymphoma, Diffuse large B-cell lymphoma, Anaplastic variant, Differential diagnosis

## Abstract

**Background:**

Primary splenic lymphoma represents a rare entity that constitutes less than 1% of non-Hodgkin lymphomas, and less than 2% of all lymphomas. Diffuse large B-cell lymphoma (DLBCL) is the most common histological subtype of primary splenic lymphomas. DLBCL encompasses a heterogeneous entity with distinct morphological variants. The anaplastic variant of DLBCL was first defined in the 2017 World Health Organization classification as a rare histological subtype that constitutes less than 3.4% of DLBCL cases.

**Case presentation:**

A 65-year-old Syrian man presented to our hospital with constant dull localized left upper quadrant abdominal pain for about 20 days accompanied by general weakness, loss of appetite, and rapid weight loss. Clinical examination revealed isolated splenomegaly and left upper abdominal tenderness. Following physical, laboratory, and radiologic examinations, the patient underwent splenectomy. Interestingly, pathological and immunohistochemical examinations of the resected spleen confirmed the diagnosis of a primary anaplastic variant of DLBCL.

**Conclusions:**

Herein, we aimed to present an unusual combination of a rare splenic neoplasm and a unique lymphoma subtype. Furthermore, we aimed to highlight the difficulties in differential diagnosis and the importance of histological and immunohistochemical examinations with clinical correlation.

## Background

Primary splenic lymphoma (PSL) represents a rare entity that constitutes less than 1% of non-Hodgkin lymphomas (NHL) and less than 2% of all lymphomas. It was defined by Dasgupta *et al.* as a lymphoma restricted to the spleen and hilar lymph nodes, with no involvement of the liver or other sites [1, 2]. Diffuse large B-cell lymphoma (DLBCL) is the most common histological subtype of both PSLs and all NHLs. DLBCL encompasses an aggressive heterogeneous entity with distinct morphological variants [3]. Recent studies have demonstrated that distinct morphological subtypes of DLBCL are associated with distinct genetic profiles. The activated B-cell-like (ABC) histological subtype was associated with the MCD genetic profile (co-occurrence of MYD88 ^L265P^ and CD79b mutations), whereas the germinal-center B-cell-like (GCB) subtype was associated with the EZB genotype (involving BCL2 translocations and EZH2 mutations). Furthermore, the unclassified histological subtype of DLBCL was found to be associated with the BN2 genotype (including NOTCH2 mutations and BCL6 fusions) [4].

The anaplastic variant of DLBCL (AV-DLBCL) is a rare histological subtype that constitutes less than 3.4% of DLBCL cases and is characterized by a sinusoidal or cohesive proliferation of large pleomorphic cells resembling the hallmark cells of anaplastic large cell lymphoma (ALCL), in addition to bizarre Reed-Sternberg-like cells. Only a few studies in the literature have discussed this unique neoplasm, due to the challenging morphological features [5]. Herein we represent an unusual case of a primary splenic anaplastic variant of DLBCL.

## Case presentation

We report the case of a 65-year-old Syrian man who presented to our hospital with constant dull localized left upper quadrant abdominal pain for about 20 days accompanied by weakness and loss of appetite with no fever, hematemesis, or vomiting. The patient had experienced rapid weight loss (approximately 20 kg in the past 2 months) with no remarkable medical history except for diabetes mellitus which was diagnosed 20 years before. Clinical examination revealed isolated splenomegaly and left upper abdominal tenderness.

**Fig. 1:**
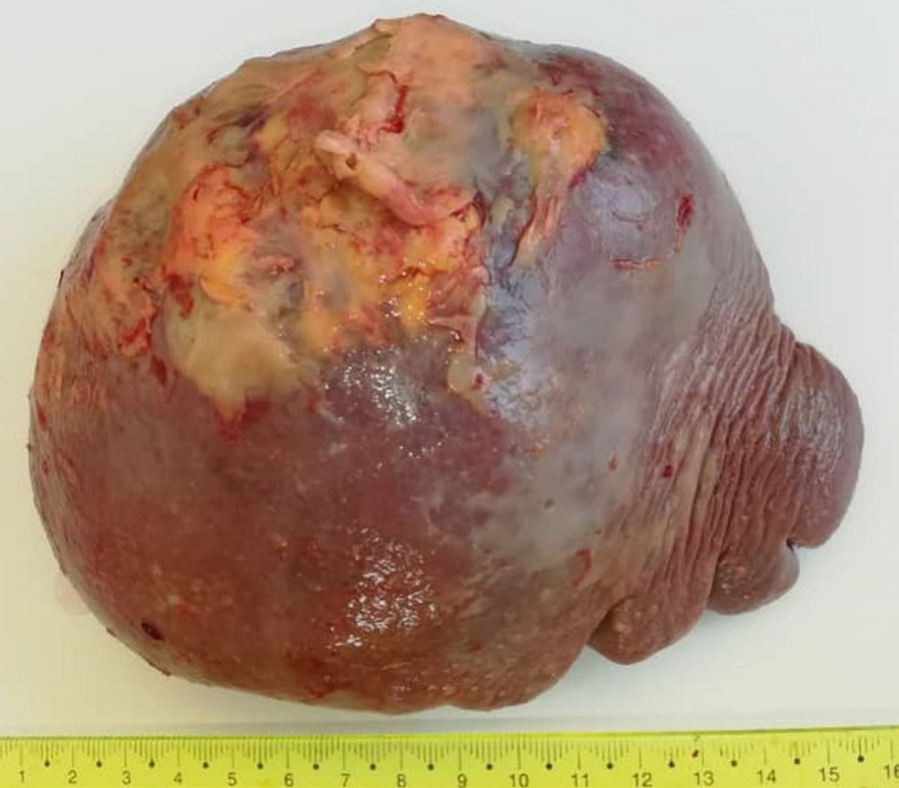
A macroscopic image demonstrating an enlarged spleen weighing 730 g and measuring 17 × 14 × 8 cm, with sclerotic necrotic foci on the surface

**Fig. 2:**
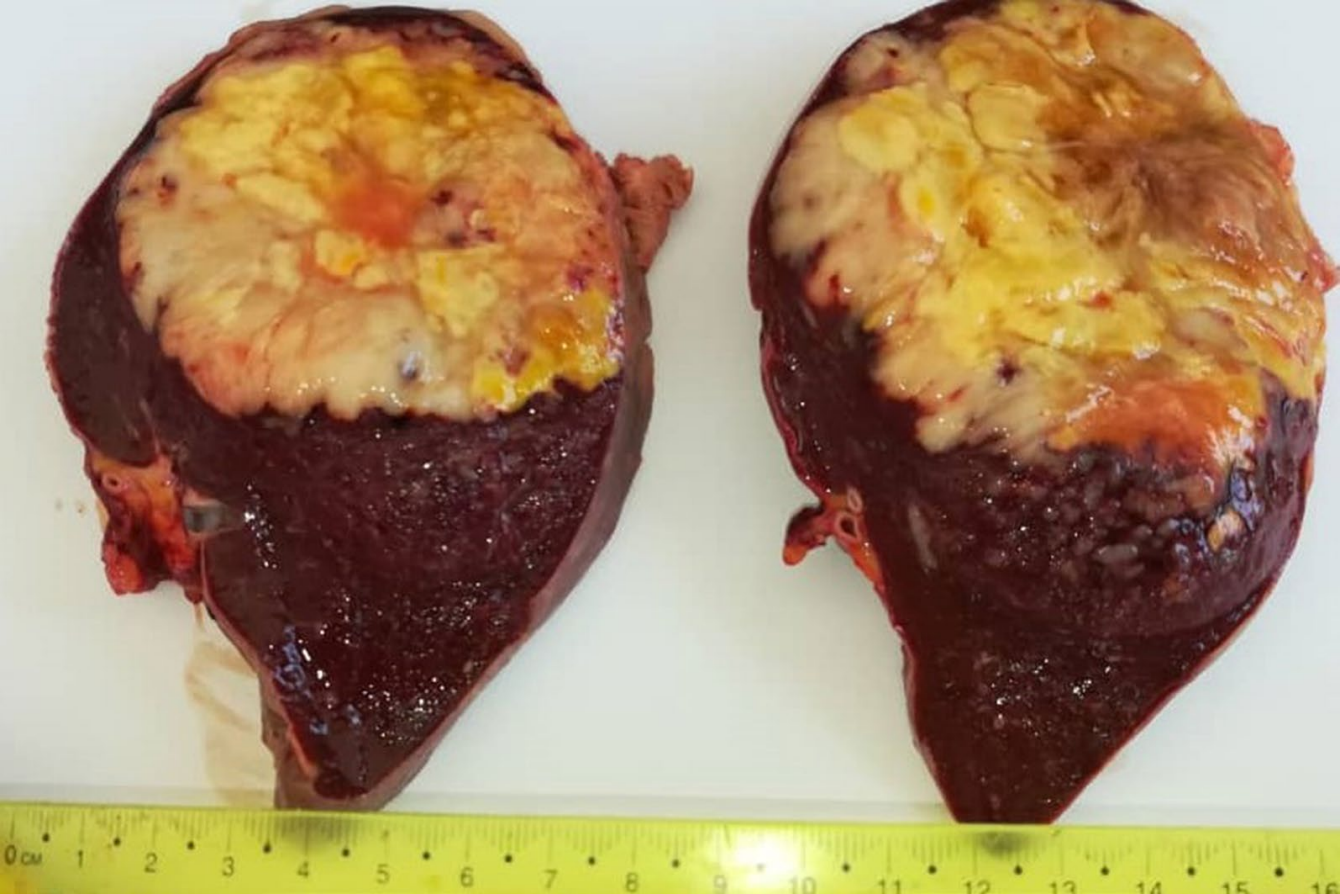
A macroscopic image of the cut section demonstrating pale yellowish soft necrotic foci replacing the splenic tissue

Laboratory examination revealed a white blood cell (WBC) count of 2.8 × 10^3^ µ/L, lymphocytes 47%, granulocytes 42.1%, red blood cell (RBC) count 2.74 x 10^6^  µ/L, hemoglobin (Hgb) 8g/dL, glucose 160 mg/dL, C-reactive protein (CRP) 63.8 mg/L, and lactate dehydrogenase (LDH) 1200 U/L. Other markers were within normal limits. Accordingly, a bone marrow biopsy was performed and revealed 50% cellularity with complete trilineage hematopoiesis and scattered mild reactive lymphoid infiltrate with no cellular atypia. Also, an upper gastrointestinal (GI) endoscopy revealed no remarkable findings. Therefore, the patient underwent splenectomy, and gross examination revealed an enlarged spleen weighing 730 g and measuring 17 × 14 × 8 cm, with sclerotic necrotic foci on the surface (Fig. 1). Cut section demonstrated pale yellowish soft necrotic foci replacing the splenic tissue (Fig. 2). Neither enlarged lymph nodes nor other lesions were detected through full-body computed tomography (CT) scan.

**Fig 3:**
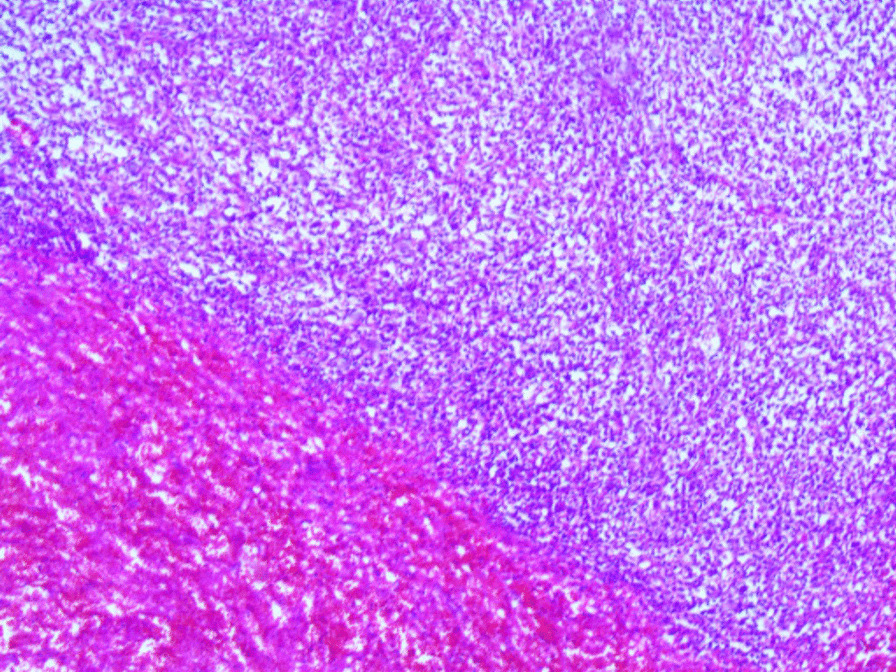
Microscopic images demonstrating the replacement of the spleen tissue by large atypical lymphoid cells, with scattered large polygonal cells characterized by bizarre pleomorphic nuclei resembling the cells of anaplastic large cell lymphoma, and fewer cells with large atypical nuclei resembling Reed-Sternberg cells. [Hematoxylin and eosin (H&E) stain, original magnification ×40]

**Fig. 4:**
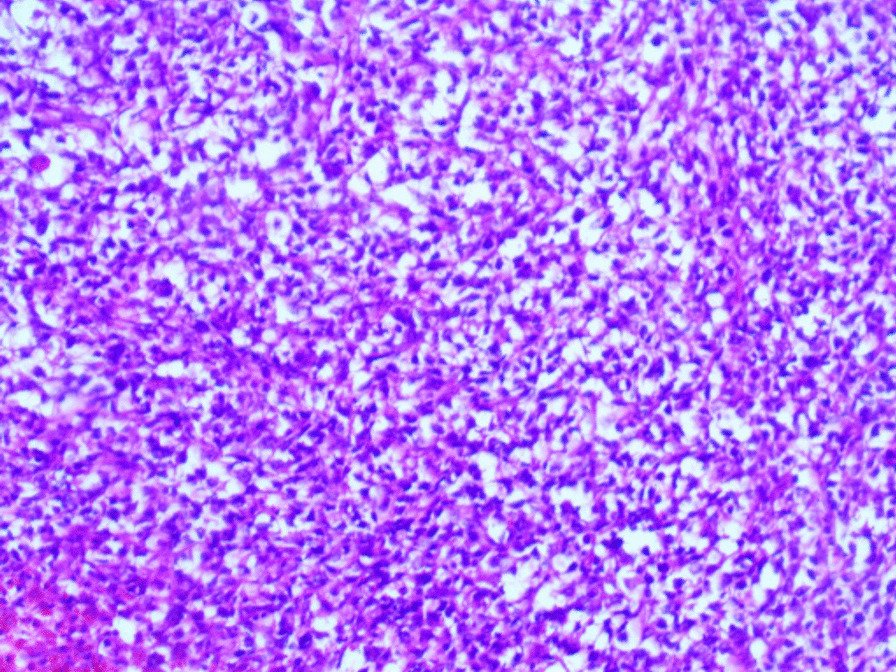
Microscopic images demonstrating the replacement of the spleen tissue by large atypical lymphoid cells, with scattered large polygonal cells characterized by bizarre pleomorphic nuclei resembling the cells of anaplastic large cell lymphoma, and fewer cells with large atypical nuclei resembling Reed-Sternberg cells. [Hematoxylin and eosin (H&E) stain, original magnification ×100]

**Fig. 5:**
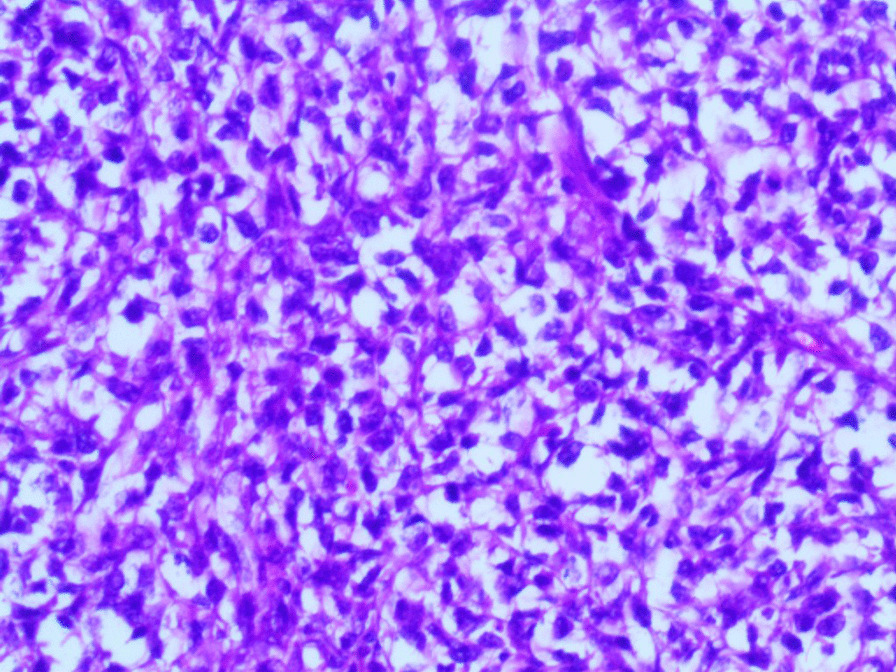
Microscopic images demonstrating the replacement of the spleen tissue by large atypical lymphoid cells, with scattered large polygonal cells characterized by bizarre pleomorphic nuclei resembling the cells of anaplastic large cell lymphoma, and fewer cells with large atypical nuclei resembling Reed-Sternberg cells. [Hematoxylin and eosin (H&E) stain, original magnification ×200]

**Fig. 6:**
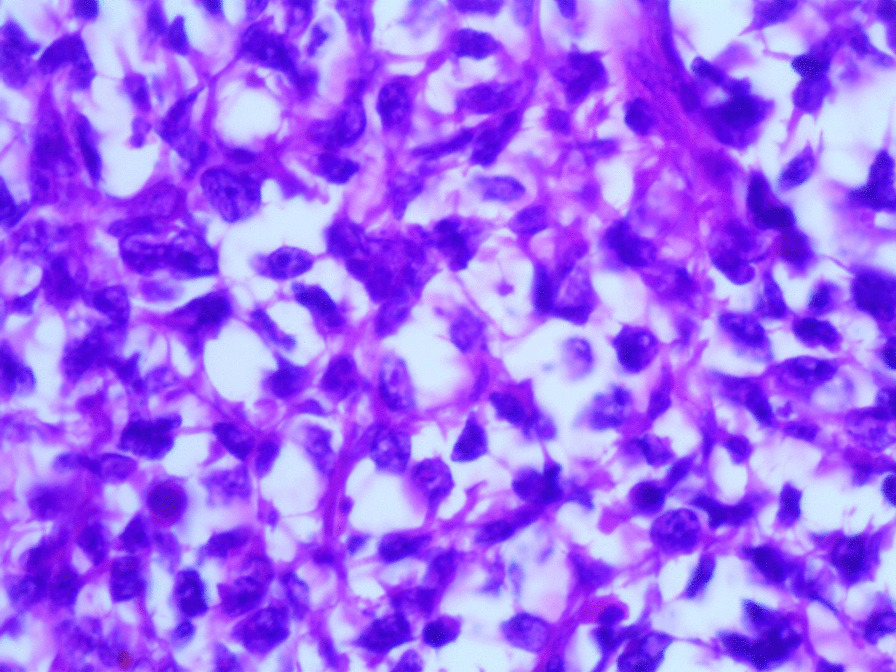
Microscopic images demonstrating the replacement of the spleen tissue by large atypical lymphoid cells, with scattered large polygonal cells characterized by bizarre pleomorphic nuclei resembling the cells of anaplastic large cell lymphoma, and fewer cells with large atypical nuclei resembling Reed-Sternberg cells. [Hematoxylin and eosin (H&E), stain original magnification ×400]

**Fig. 7:**
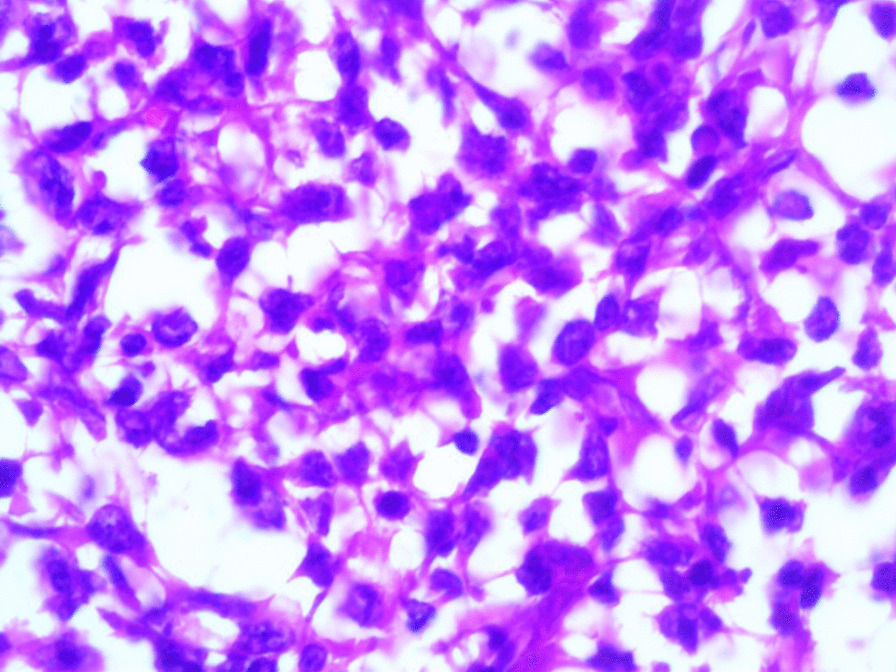
Microscopic images demonstrating the replacement of the spleen tissue by large atypical lymphoid cells, with scattered large polygonal cells characterized by bizarre pleomorphic nuclei resembling the cells of anaplastic large cell lymphoma, and fewer cells with large atypical nuclei resembling Reed-Sternberg cells. [Hematoxylin and eosin (H&E), stain original magnification ×400]

**Fig. 8:**
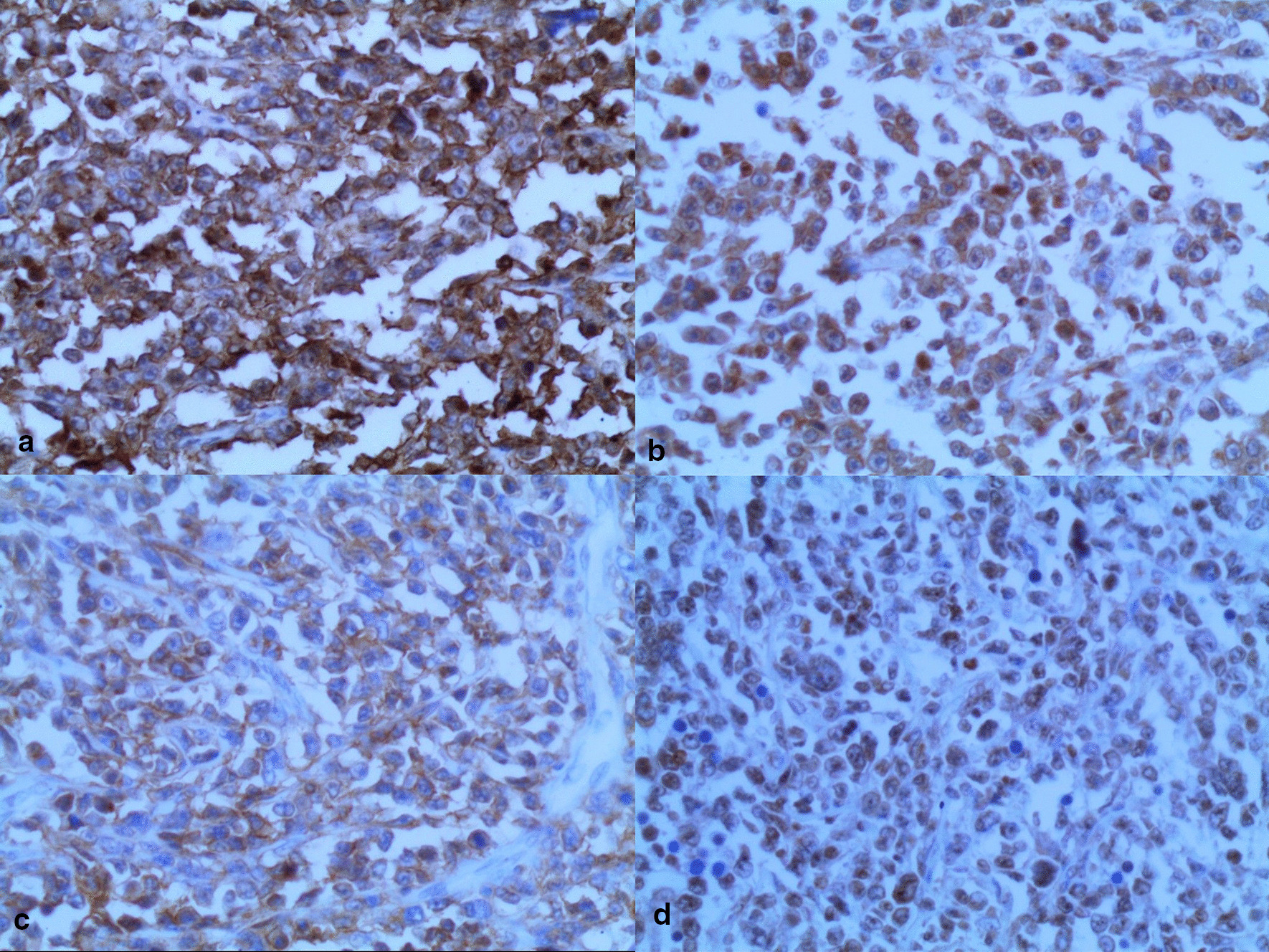
Immunohistochemistry of the neoplasm: **a** High positivity for CD20. **b** High positivity for CD30. **c** Positivity for CD45. **d** Positivity for BCL2

**Fig. 9:**
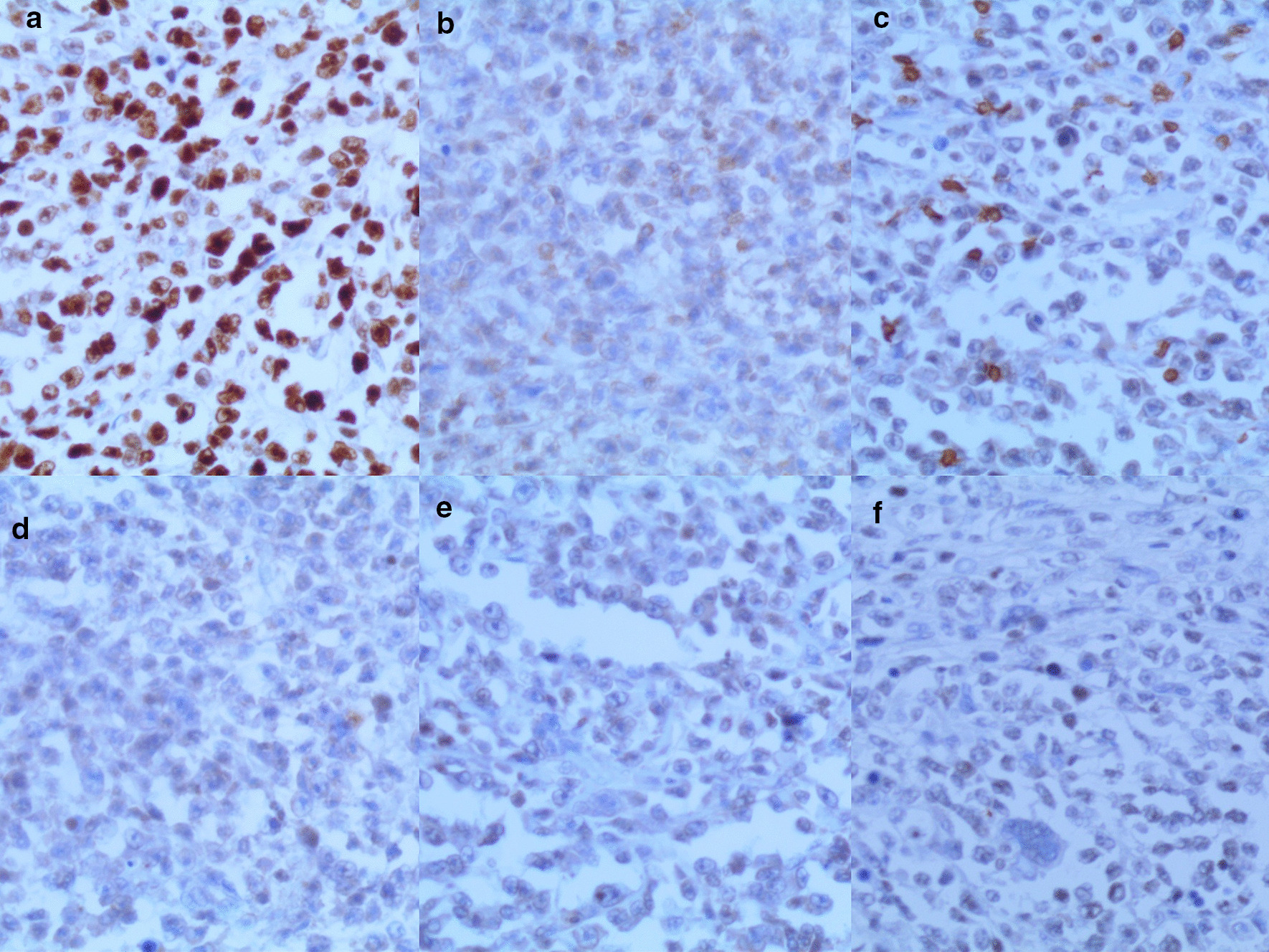
Immunohistochemistry of the neoplasm: **a** Positivity for Ki-67. **b** CD5 is negative in the neoplastic cells. **c** CD3 is negative in the neoplastic cells. **d** CD10 is negative in the neoplastic cells. **e** CD15 is negative in the neoplastic cells. **f** ALK-1 is negative in the neoplastic cells)

Microscopic examination revealed the replacement of the spleen tissue by large atypical lymphoid cells, with scattered large polygonal cells characterized by bizarre pleomorphic nuclei resembling the cells of ALCL, and fewer cells with large atypical nuclei resembling Reed-Sternberg cells (Figs. 3, 4, 5, 6, 7). Accordingly, primary differential diagnoses included ALCL, DLBCL, and classical Hodgkin lymphoma (CHL). Subsequently, immunohistochemical staining revealed strong positivity for CD30, CD45, and CD20, whereas CD3, CD5, CD10, CD15, and ALK-1 were negative, and BCL2 showed a focal weak positivity (Figs. 8, 9). Therefore, the final diagnosis was confirmed by three pathologists at our department as AV-DLBCL. The patient was discharged 9 days after hospitalization and was scheduled for chemotherapy treatment including cyclophosphamide, vincristine, prednisone, and rituximab (R-CVP).

## Discussion

Although secondary involvement of the spleen is a common finding in nodal and extranodal lymphomas, primary lymphomas of the spleen represent an extremely rare entity. A strict diagnostic criterion is essential to establish the diagnosis including splenomegaly caused by a lymphoma restricted to the spleen with no involvement of the liver or other sites [2]. Most patients present with nonspecific symptoms including left upper abdominal quadrant pain, general weakness, and loss of appetite, as in our case, in addition to symptoms associated with invasion or compression of the adjacent organs [6, 7]. Furthermore, medical history might play an essential role in the progression of symptoms. In 1998, Nai *et al.* reported an interesting case of a 61-year-old woman who developed a rapid thrombocytopenia and splenomegaly before being diagnosed with a primary splenic ALCL. Nevertheless, the patient had a history of chronic lymphocytic leukemia (CLL) which was suspected as the main cause of the rapid progression of symptoms [8]. In our case, medical history was unremarkable except for diabetes mellitus, and no rapid progression was reported.

Tumor staging plays a crucial role in the prognosis and management of splenic lymphomas. According to Ahmann *et al.*, splenic lymphomas are classified into three stages: stage I, which refers to the strict involvement of the spleen; stage II, which refers to hilar lymph node involvement; and stage III, which refers to extra-splenic involvement [9]. Accordingly, our case was classified as stage I. Furthermore, an interesting case series of primary splenic DLBCL in 2007 demonstrated signs of poor prognosis including elevated serum LDH levels as in our case, high proliferation rate, the presence of B symptoms, and neoplasm size greater than 10 cm [10].

PSLs encompass various histological subtypes including DLBCL, marginal zone lymphomas, ALCL, and follicular lymphomas [11]. The most common histological variant is DLBCL, which also represents the most common lymphoid malignancy, accounting for approximately 31% of all NHL cases [12].

DLBCL was classified into four morphological categories according to the fourth edition of the World Health Organization (WHO) classification in 2008: (a) DLBCL—not otherwise specified (DLBCL, NOS), which constitutes the most common variant, (b) DLBCL with predominant extranodal location, (c) large cell lymphoma of terminally differentiated B cells, and (d) borderline cases. [13, 14]

AV-DLBCL was first defined in the 2017 WHO classification as a rare morphological variant of DLBCL-NOS. AV-DLBCL constitutes less than 3.4% of all DLBCL cases and tends to affect all ages, with a slight male predominance [15, 16]. AV-DLBCL might affect nodal and extranodal sites, whereas it is extremely rare to be primarily diagnosed in the spleen. Patients usually present with B symptoms including fever, weight loss, and night sweats [5, 15, 16]

Microscopically, AV-DLBCL is characterized by a sinusoidal or cohesive proliferation of large, pleomorphic, bizarre-shaped neoplastic cells with irregularly shaped nuclei, coarse chromatin, and one or more eosinophilic nucleoli. The nuclei might have a horseshoe-like or kidney-shaped form which resembles the characteristic neoplastic cells of ALCL. In addition to these cells, bizarre Reed-Sternberg-like tumor cells and binucleated or multinucleated tumor cells can be found. Although there is no specific marker to confirm the diagnosis, neoplastic cells show strong positivity for CD30 and B-cell markers including CD20 as in our case, in addition to the negativity for anaplastic lymphoma kinase (ALK) and T-cell markers [16, 17].

The difficulties in differential diagnosis represent a major challenge due to the overlapping morphological features in addition to the lack of specific diagnostic criteria. The main differential diagnoses include ALCL, ALK-positive large B-cell lymphoma (ALK+ LBCL), and CHL. ALCL is defined as a CD30-positive neoplasm of T- or null-cell lineage, characterized by the proliferation of large pleomorphic cells with kidney-shaped nuclei. The negative expression of T-cell markers (CD5, CD3) in our case, in addition to the strong expression of the B-cell marker (CD20), was crucial to exclude ALCL [16, 18, 19].

Furthermore, ALK+ LBCL is a rare subgroup of DLBCL characterized by diffuse infiltration of medium to large neoplastic cells with round centric or eccentric nuclei, and is usually negative for CD30 and CD20 with a strong positivity to ALK, in contrast to the findings of our case [16, 18, 20]. Also, the negative expression of CD15 in addition to the absence of inflammatory background and band-like fibrosis was essential to exclude CHL [15, 16].

In addition to the aforementioned neoplasms, splenic localization superimposed further differential diagnosis considerations, including splenic marginal zone lymphoma (SMZL), which was excluded by the CD30 positivity, and splenic follicular lymphoma (SFL), which was excluded by the negativity for CD10 [6, 11, 13].

On the molecular genetics level, according to a study reported by Li *et al.*, AV-DLBCL shows a high frequency of the TP53 mutation, P53 positivity, and concurrent abnormalities of MYC, BCL2, and BCL6 rearrangements. In our case, BCL2 revealed a focal positivity in the immunohistochemical examination [21]. Nevertheless, other techniques were not available at our department, and the diagnosis was based on detailed histological and immunohistochemical examinations by three pathologists.

Treatment decision represents a dilemma due to the lack of a standard regimen. Nevertheless, splenectomy is considered the first-line procedure and has shown high efficacy in both diagnosis and treatment of splenic lesions as in our case. Furthermore, several studies have demonstrated the role of splenectomy in improving the prognosis for PSLs. Adjuvant chemotherapy including R-CHOP regimen (rituximab, cyclophosphamide, doxorubicin, vincristine, and prednisone) or DA-EPOCH-R regimen (etoposide, prednisone, vincristine, cyclophosphamide, doxorubicin, and rituximab) might improve survival following splenectomy [22–24]. In our case, R-CVP was scheduled in order to minimize the side effects of doxorubicin.

## Conclusions

In conclusion, diagnosing AV-DLBCL in the spleen is a significant challenge due to the difficulties in differential diagnosis with ALCL and other lymphoma subtypes. AV-DLBCL can be distinguished from ALCL by their immune features of B-cell lineage and negativity of T-cell markers and ALK-1 expression. Nevertheless, with an accurate clinical approach with proper pathological and immunohistochemical correlations, the diagnosis was confirmed. Furthermore, we managed to present an unusual combination of a rare splenic neoplasm and a unique lymphoma subtype.

## Data Availability

Not applicable.

## Abbreviations

PSL: Primary splenic lymphoma
DLBCL: Diffuse large B-cell lymphoma
AV-DLBCL: Anaplastic variant of diffuse large B-cell Lymphoma
ALK: Anaplastic lymphoma kinase
CHL: Classical Hodgkin lymphoma
NHL: Non-Hodgkin lymphoma
CD: Cluster of differentiation
H&E: Hematoxylin and eosin
CT: Computed tomography
WBC: White blood cells
RBC: Red blood cells
Hgb: Hemoglobin
CRP: C-Reactive protein
LDH: Lactate dehydrogenase
